# Coding-Complete Genome Sequences of Alpha and Delta SARS-CoV-2 Variants from Kamphaeng Phet Province, Thailand, from May to July 2021

**DOI:** 10.1128/MRA.00877-21

**Published:** 2021-12-02

**Authors:** Kanyarat Phutthasophit, Darunee Buddhari, Piyawan Chinnawirotpisan, Khajohn Joonlasak, Wudtichai Manasatienkij, Angkana Huang, Thitiman Kaewkao, Narong Mahayos, Rotjana Khontong, Sopon Iamsirithaworn, Anthony R. Jones, Aaron R. Farmer, Stefan Fernandez, Chonticha Klungthong

**Affiliations:** a Department of Virology, U.S. Army Medical Directorate, Armed Forces Research Institute of Medical Sciences, Bangkok, Thailand; b Kamphaeng Phet Provincial Hospital, Ministry of Public Health, Kamphaeng Phet, Thailand; c Department of Disease Control, Ministry of Public Health, Talard-Kwan, Nonthaburi, Thailand; DOE Joint Genome Institute

## Abstract

We report coding-complete genome sequences of 44 severe acute respiratory syndrome coronavirus 2 (SARS-CoV-2) strains of the alpha and delta variants identified from patients in Kamphaeng Phet, Thailand. Two nonsense mutations in open reading frame 3a (ORF3a) (G254*) and ORF8 (K68*) were found in the alpha variant sequences. Two lineages of the delta variant, B.1.617.2 and AY.30, were found.

## ANNOUNCEMENT

Severe acute respiratory syndrome coronavirus 2 (SARS-CoV-2), belonging to the *Coronaviridae* family and the *Betacoronavirus* genus, has been reported in Kamphaeng Phet province (KPP), Thailand, since 23 December 2020 (1). Surveillance of viral genetic variation provides information that could be useful for developing a prevention and control plan.

A total of 44 viral RNA samples extracted from nasopharyngeal/oropharyngeal swab specimens from SARS-CoV-2 patients under investigation and general population surveillance among subjects with unknown coronavirus disease 2019 (COVID-19) vaccination status were tested. These samples included samples from high-risk close contacts of positive cases and active cases found in an outbreak area from 3 May 2021 to 18 July 2021. This work was performed under a Walter Reed Army Institute of Research (WRAIR) public health research/nonresearch determination (WRAIR number 2741) to support core public health functions. The investigators have adhered to the policies for protection of human subjects as prescribed in publication AR 70-25.

Samples were extracted with the QIAamp viral RNA minikit (Qiagen) and MagCore nucleic acid extraction kit and sequenced by the ARTIC protocol with v3 primers (2). DNA library preparation and sequencing were performed with a DNA library preparation kit and the MiSeq reagent kit v2 (2 × 250 nucleotides), respectively. Two sequencing runs (22 samples/run) were performed on the Illumina MiSeq platform. Sequence reads from 94% of the clusters, with Phred quality (Q) scores of ≥30, were analyzed. Bioinformatic methods were described in previous reports (3, 4) and in Table 1. These methods included the Burrows-Wheeler Aligner (BWA) MEM algorithm (5), which was used for sequence mapping with the Wuhan-Hu-1 genome (GenBank accession number NC_045512.2), and iVar v1.2.2 (6) and SAMtools (7), which were used for primer region trimming and variant calling (Q scores of ≥25), respectively. Consensus sequences were generated using iVar v1.2.2 (Q scores of ≥25 and depth of coverage [DOC] of ≥10×). Ambiguous bases, deletions, and gaps were identified and confirmed by genome-guided assembly with the reference sequence using Trinity v2.8.5 (8) and Sanger sequencing. Pangolin v3.1.14 with lineages version 2021-09-28 (9), GISAID clade nomenclature (10), and phylogenetic analysis (11–13) were used to determine SARS-CoV-2 lineages. Nextclade v1.6.0 (14) was used to identify variants. All tools were run with default parameters.

**TABLE 1:** Sequence data for 44 SARS-CoV-2 sequences obtained from this study^*a*^

Sequence identifier	GenBank accession no.	SRA accession no.	Collection date	Nextstrain clade	Pangolin lineage (v2021-09-28)	No. of raw paired-end reads	GC content (%)	Length of consensus sequence of coding region (bp)	Mean DOC (×)	Breadth of coverage (10× genome coverage) (%)	Amino acid substitutions^*b*^
SARS-CoV-2/Thailand/AFRIMS-COV0087	MZ888515	SRR15571425	3-May-2021	20I (alpha, V1)	B.1.1.7	792,232	38.4	29,390	4,782	97.2	ORF1a: L2780F; ORF1b: R1383K, L2687I, I2689S, S2690D, C2691V, R2692L, S2693V, V2694N, L2695N; ORF3a: T14I, F15L, G254*; ORF8: K68*; ORF10: P10S
SARS-CoV-2/Thailand/AFRIMS-COV0175	MZ888516	SRR15571424	17-May-2021	20I (alpha, V1)	B.1.1.7	1,402,758	38.5	29,390	8,622	98.1	ORF1a: L2780F; ORF1b: R1383K, L2687I, I2689S, S2690D, C2691V, R2692L, S2693V, V2694N, L2695N; ORF3a: F15L, G254*; ORF8: K68*; ORF10: P10S
SARS-CoV-2/Thailand/AFRIMS-COV0178	MZ888517	SRR15571413	17-May-2021	20I (alpha, V1)	B.1.1.7	957,772	38.7	29,390	5,298	97.3	ORF1a: L2780F; ORF1b: R1383K, L2687I, I2689S, S2690D, C2691V, R2692L, S2693V, V2694N, L2695N; ORF3a: F15L, G254*; ORF8: K68*; ORF10: P10S
SARS-CoV-2/Thailand/AFRIMS-COV0194	MZ888518	SRR15571402	17-May-2021	20I (alpha, V1)	B.1.1.7	886,654	38.4	29,390	5,236	97.9	ORF1a: L2780F; ORF1b: R1383K, L2687I, I2689S, S2690D, C2691V, R2692L, S2693V, V2694N, L2695N; ORF3a: F15L, G254*; ORF8: K68*; ORF10: P10S
SARS-CoV-2/Thailand/AFRIMS-COV0195	MZ888519	SRR15571391	17-May-2021	20I (alpha, V1)	B.1.1.7	889,804	38.4	29,390	5,482	98.5	ORF1a: L2780F; ORF1b: R1383K, L2687I, I2689S, S2690D, C2691V, R2692L, S2693V, V2694N, L2695N; ORF3a: F15L, G254*; ORF8: K68*; ORF10: P10S
SARS-CoV-2/Thailand/AFRIMS-COV0196	MZ888520	SRR15571386	17-May-2021	20I (alpha, V1)	B.1.1.7	931,994	38.4	29,390	5,779	97.9	ORF1a: L2780F; ORF1b: R1383K, L2687I, I2689S, S2690D, C2691V, R2692L, S2693V, V2694N, L2695N; ORF3a: F15L, G254*; ORF8: K68*; ORF10: P10S
SARS-CoV-2/Thailand/AFRIMS-COV0413	MZ888521	SRR15571385	28-May-2021	20I (alpha, V1)	B.1.1.7	918,084	38.5	29,390	5,536	97.2	ORF1a: L2780F; ORF1b: R1383K, L2687I, I2689S, S2690D, C2691V, R2692L, S2693V, V2694N, L2695N; ORF3a: F15L, G254*, V273M; ORF8: K68*; ORF10: P10S
SARS-CoV-2/Thailand/AFRIMS-COV0480	MZ888522	SRR15571384	3-Jun-2021	20I (alpha, V1)	B.1.1.7	799,474	38.7	29,390	4,428	92.3	ORF1a: M1586I, L2780F; ORF1b: R1383K, L2687I, I2689S, S2690D, C2691V, R2692L, S2693V, V2694N, L2695N; ORF3a: F15L, G254*; ORF8: K68*; ORF10: P10S
SARS-CoV-2/Thailand/AFRIMS-COV0533	MZ888523	SRR15571383	5-Jun-2021	20I (alpha, V1)	B.1.1.7	910,276	38.2	29,390	5,505	99.3	ORF1a: L2780F; ORF1b: R1383K, L2687I, I2689S, S2690D, C2691V, R2692L, S2693V, V2694N, L2695N; ORF3a: F15L, G254*, V273M; ORF8: L60F, K68*; ORF10: P10S
SARS-CoV-2/Thailand/AFRIMS-COV0609	MZ888524	SRR15571382	10-Jun-2021	20I (alpha, V1)	B.1.1.7	1,150,826	38.3	29,390	6,903	99.6	ORF1a: L2780F, P3359S; ORF1b: R1383K, L2687I, I2689S, S2690D, C2691V, R2692L, S2693V, V2694N, L2695N; ORF3a: F15L, G254*, V273M; ORF8: K68*; ORF10: P10S
SARS-CoV-2/Thailand/AFRIMS-COV0654	MZ888525	SRR15571423	11-Jun-2021	20I (alpha, V1)	B.1.1.7	956,642	38.2	29,390	5,868	99.5	ORF1a: L2780F, P3359S; ORF1b: R1383K, L2687I, I2689S, S2690D, C2691V, R2692L, S2693V, V2694N, L2695N; ORF3a: F15L, G254*, V273M; ORF8: K68*; ORF10: P10S
SARS-CoV-2/Thailand/AFRIMS-COV0804	MZ888526	SRR15571422	15-Jun-2021	20I (alpha, V1)	B.1.1.7	841,232	38.2	29,390	5,067	99.5	ORF1a: L2780F, P3359S; ORF1b: R1383K, L2687I, I2689S, S2690D, C2691V, R2692L, S2693V, V2694N, L2695N; ORF3a: F15L, G254*, V273M; ORF8: K68*; ORF10: P10S
SARS-CoV-2/Thailand/AFRIMS-COV0894	MZ888527	SRR15571421	17-Jun-2021	20I (alpha, V1)	B.1.1.7	1,131,734	38.3	29,390	6,310	99.7	ORF1a: L2780F, P3359S; ORF1b: R1383K, L2687I, I2689S, S2690D, C2691V, R2692L, S2693V, V2694N, L2695N; ORF3a: F15L, G254*, V273M; ORF8: K68*; ORF10: P10S
SARS-CoV-2/Thailand/AFRIMS-COV1003	MZ888528	SRR15571420	18-Jun-2021	20I (alpha, V1)	B.1.1.7	785,178	38.2	29,390	4,605	99.1	ORF1a: L2780F, L3330S; ORF1b: R1383K, H2571Y, L2687I, I2689S, S2690D, C2691V, R2692L, S2693V, V2694N, L2695N; ORF3a: F15L, G254*; E: L73F; ORF8: K68*; ORF10: P10S
SARS-CoV-2/Thailand/AFRIMS-COV1099	MZ888529	SRR15571419	20-Jun-2021	20I (alpha, V1)	B.1.1.7	878,732	38.8	29,390	5,429	97.3	ORF1a: L2780F, V3690L; ORF1b: R1383K, L2687I, I2689S, S2690D, C2691V, R2692L, S2693V, V2694N, L2695N; ORF3a: F15L, G254*; ORF8: K68*; ORF10: P10S
SARS-CoV-2/Thailand/AFRIMS-COV1118	MZ888530	SRR15571418	20-Jun-2021	20I (alpha, V1)	B.1.1.7	808,026	38.2	29,390	4,886	99.5	ORF1a: L2780F; ORF1b: S759G, R1383K, L2687I, I2689S, S2690D, C2691V, R2692L, S2693V, V2694N, L2695N; ORF3a: F15L, G254*; ORF8: K68*; ORF10: P10S
SARS-CoV-2/Thailand/AFRIMS-COV1137	MZ888531	SRR15571417	20-Jun-2021	20I (alpha, V1)	B.1.1.7	1,411,774	38.1	29,390	8,642	99.6	ORF1a: E1377G, L2780F; ORF1b: S759G, R1383K, L2687I, I2689S, S2690D, C2691V, R2692L, S2693V, V2694N, L2695N; ORF3a: F15L, G254*; ORF8: K68*; ORF10: P10S
SARS-CoV-2/Thailand/AFRIMS-COV1365	MZ888532	SRR15571416	24-Jun-2021	21A (delta)	AY.30	1,112,760	38.3	29,396	6,569	98.7	ORF1b: F1504L; N: L139F
SARS-CoV-2/Thailand/AFRIMS-COV1370	MZ888533	SRR15571415	24-Jun-2021	21A (delta)	AY.30	809,794	38.2	29,396	4,798	98.5	ORF1b: F1504L; N: L139F
SARS-CoV-2/Thailand/AFRIMS-COV1380	MZ888534	SRR15571414	24-Jun-2021	21A (delta)	AY.30	857,702	38.3	29,396	5,099	98.8	ORF1b: F1504L; N: L139F
SARS-CoV-2/Thailand/AFRIMS-COV1385	MZ888535	SRR15571412	24-Jun-2021	21A (delta)	AY.30	975,240	38.1	29,396	6,027	97.9	ORF1b: F1504L; N: L139F
SARS-CoV-2/Thailand/AFRIMS-COV1392	MZ888536	SRR15571411	24-Jun-2021	20I (alpha, V1)	B.1.1.7	901,192	38.1	29,390	5,143	94.5	ORF1a: L2780F, P3504L, L3829F; ORF1b: R1383K, L2687I, I2689S, S2690D, C2691V, R2692L, S2693V, V2694N, L2695N; S: P809S; ORF3a: F15L, G254*; ORF8: K68*; N: L230F; ORF10: P10S
SARS-CoV-2/Thailand/AFRIMS-COV1515	MZ888537	SRR15571410	2-Jul-2021	21A (delta)	B.1.617.2	1,094,580	38.4	29,396	7,475	99.7	ORF1a: E148G, L309P, A1306S, L1640P, P2046L, P2287S, V2930L, V3209A, T3255I, T3646A; ORF1b: F1504L, Y2285H; ORF3a: L140F; E: V62F; ORF7a: F116L; ORF7b: T40I; N: K385R
SARS-CoV-2/Thailand/AFRIMS-COV1530	MZ888538	SRR15571409	3-Jul-2021	21A (delta)	B.1.617.2	746,370	38.1	29,396	4,909	97.0	ORF1a: E148G, L309P, K1230N, A1306S, L1640P, P2046L, Y2092H, P2287S, V2930L, V3209A, T3255I, T3646A; ORF1b: F1504L, D1869Y, Y2285H, D2429Y; ORF3a: L140F; E: V62F; ORF7a: F116L; ORF7b: T40I; N: K385R
SARS-CoV-2/Thailand/AFRIMS-COV1538	MZ888539	SRR15571408	4-Jul-2021	21A (delta)	B.1.617.2	857,404	38.3	29,396	5,732	99.6	ORF1a: E148G, L309P, A1306S, L1640P, P2046L, P2287S, V2930L, V3209A, T3255I, T3646A; ORF1b: F1504L, Y2285H; ORF3a: L140F; E: V62F; ORF7a: F116L; ORF7b: T40I; N: K385R
SARS-CoV-2/Thailand/AFRIMS-COV1588	MZ888540	SRR15571407	5-Jul-2021	21A (delta)	AY.30	895,954	38.3	29,396	6,057	99.3	ORF1b: F1504L; N: L139F
SARS-CoV-2/Thailand/AFRIMS-COV1677	MZ888541	SRR15571406	7-Jul-2021	21A (delta)	B.1.617.2	554,830	38.9	29,396	3,636	94.3	ORF1a: E148G, L309P, A1306S, L1640P, P2046L, Y2092H, P2287S, V2930L, V3209A, T3255I, T3646A; ORF1b: F1504L, D1869Y, A1918V, Y2285H; ORF3a: L140F; E: V62F; ORF7a: F116L; ORF7b: T40I; N: K385R
SARS-CoV-2/Thailand/AFRIMS-COV1772	MZ888542	SRR15571405	8-Jul-2021	21A (delta)	AY.30	828,630	38.6	29,396	5,479	97.4	ORF1b: F1504L, Q2615R; N: L139F
SARS-CoV-2/Thailand/AFRIMS-COV1802	MZ888543	SRR15571404	9-Jul-2021	21A (delta)	AY.30	873,914	38.4	29,396	5,917	99.6	ORF1b: F1504L; N: L139F
SARS-CoV-2/Thailand/AFRIMS-COV1865	MZ888544	SRR15571403	10-Jul-2021	21A (delta)	B.1.617.2	818,330	38.5	29,396	5,521	95.2	ORF1a: E148G, L309P, A1049V, A1306S, L1640P, P2046L, Y2092H, P2287S, V2930L, V3209A, T3255I, T3646A; ORF1b: F1504L, D1869Y, Y2285H; ORF3a: L140F; E: V62F; ORF7a: F116L; ORF7b: T40I; N: K385R
SARS-CoV-2/Thailand/AFRIMS-COV1904	MZ888545	SRR15571401	11-Jul-2021	21A (delta)	AY.30	748,000	38.7	29,396	4,954	96.2	ORF1b: F1504L; ORF3a: W45L; N: L139F
SARS-CoV-2/Thailand/AFRIMS-COV1956	MZ888546	SRR15571400	11-Jul-2021	21A (delta)	AY.30	1,057,648	38.8	29,387	7,022	96.4	ORF1a: T3058I; ORF1b: F1504L; ORF8: I76F; N: L139F
SARS-CoV-2/Thailand/AFRIMS-COV2000	MZ895505	SRR15571399	12-Jul-2021	21A (delta)	B.1.617.2	913,670	38.9	29,402	6,061	94.0	ORF1a: E148G, L309P, A1306S, L1640P, P2046L, Y2092H, P2287S, V2930L, V3209A, T3255I, T3646A; ORF1b: F1504L, Y2285H; ORF3a: L140F; E: V62F; ORF7a: F116L; ORF7b: T40I; N: K385R
SARS-CoV-2/Thailand/AFRIMS-COV2041	MZ888547	SRR15571398	12-Jul-2021	21A (delta)	B.1.617.2	1,049,376	38.8	29,396	7,009	94.3	ORF1a: E148G, L309P, A1306S, L1640P, P2046L, P2287S, V2930L, V3209A, T3255I, T3646A; ORF1b: F1504L, Y2285H; ORF3a: L140F; E: V62F; ORF7a: F116L; ORF7b: T40I; N: K385R
SARS-CoV-2/Thailand/AFRIMS-COV2095	MZ888548	SRR15571397	13-Jul-2021	21A (delta)	AY.30	894,862	38.7	29,396	5,961	95.7	ORF1b: F1504L; N: L139F
SARS-CoV-2/Thailand/AFRIMS-COV2136	MZ888549	SRR15571396	13-Jul-2021	21A (delta)	AY.30	828,484	38.1	29,396	5,588	99.5	ORF1a: A583V; ORF1b: T284I, F1504L; N: L139F
SARS-CoV-2/Thailand/AFRIMS-COV2199	MZ888550	SRR15571395	14-Jul-2021	21A (delta)	AY.30	816,984	38.1	29,396	5,491	97.0	ORF1b: F1504L; S: A845S; N: L139F
SARS-CoV-2/Thailand/AFRIMS-COV2228	MZ888551	SRR15571394	14-Jul-2021	21A (delta)	AY.30	719,982	38.4	29,396	4,760	99.6	ORF1a: A583V; ORF1b: T284I, F1504L; N: L139F
SARS-CoV-2/Thailand/AFRIMS-COV2278	MZ888552	SRR15571393	15-Jul-2021	21A (delta)	AY.30	935,648	38.2	29,396	6,138	99.5	ORF1a: G519S; ORF1b: F1504L; N: L139F
SARS-CoV-2/Thailand/AFRIMS-COV2353	MZ888553	SRR15571392	16-Jul-2021	21A (delta)	AY.30	945,554	38.8	29,396	6,348	95.8	ORF1a: A540V, H1067Y; ORF1b: F1504L; ORF3a: W131C; N: L139F
SARS-CoV-2/Thailand/AFRIMS-COV2447	MZ888554	SRR15571390	16-Jul-2021	21A (delta)	B.1.617.2	687,846	38.3	29,396	4,546	96.6	ORF1a: E148G, L309P, A1306S, L1640P, P2046L, Y2092H, P2287S, V2930L, V3209A, T3255I, T3646A; ORF1b: A576V, F1504L, A1918V, Y2285H; S: S477I; ORF3a: L140F; E: V62F; ORF7a: P45L, F116L; ORF7b: T40I; N: K385R
SARS-CoV-2/Thailand/AFRIMS-COV2483	MZ888555	SRR15571389	17-Jul-2021	20I (alpha, V1)	B.1.1.7	885,048	38.8	29,390	5,822	97.0	ORF1a: M1586I, L2780F; ORF1b: R1383K, L2687I, I2689S, S2690D, C2691V, R2692L, S2693V, V2694N, L2695N; ORF3a: F15L, G254*; ORF8: K68*; ORF10: P10S
SARS-CoV-2/Thailand/AFRIMS-COV2513	MZ888556	SRR15571388	17-Jul-2021	21A (delta)	AY.30	861,976	38.3	29,396	5,724	98.5	ORF1b: F1504L; S: V1122L; N: L139F
SARS-CoV-2/Thailand/AFRIMS-COV2543	MZ888557	SRR15571387	18-Jul-2021	20I (alpha, V1)	B.1.1.7	845,954	38.7	29,390	5,586	97.3	ORF1a: M1586I, L2780F; ORF1b: R1383K, L2687I, I2689S, S2690D, C2691V, R2692L, S2693V, V2694N, L2695N; ORF3a: F15L, G254*; ORF8: K68*; ORF10: P10S

a The BWA MEM algorithm (5) was used for sequence mapping and assembly with the Wuhan-Hu-1 genome (GenBank accession number NC_045512.2). iVar v1.2.2 (6) and SAMtools (7) were used for primer region trimming and variant calling (Q scores of ≥25), respectively. Consensus sequences were generated using iVar v1.2.2 (Q scores of ≥25 and DOC of ≥10×). Ambiguous bases, deletions, and gaps were identified and confirmed by genome-guided assembly with the reference sequence using Trinity v2.8.5 (8) and Sanger sequencing. Pangolin v3.1.14 with lineages version 2021-09-28 (9), GISAID clade nomenclature (10), and phylogenetic analysis (11–13) were used to determine SARS-CoV-2 lineages. Nextclade v1.6.0 (14) was used to identify variants. All tools were run with default parameters.

b Nucleotide and amino acid substitutions and annotation were analyzed using an in-house bioinformatics pipeline (19). All alpha variant sequences were aligned with the first alpha variant sequence collected in Thailand (GISAID accession number EPI_ISL_1346626), which was collected on 21 December 2020. All delta variant sequences were aligned with the first delta variant sequence collected in Thailand (GISAID accession number EPI_ISL_2104743), which was collected on 2 May 2021.

**FIG 1:**
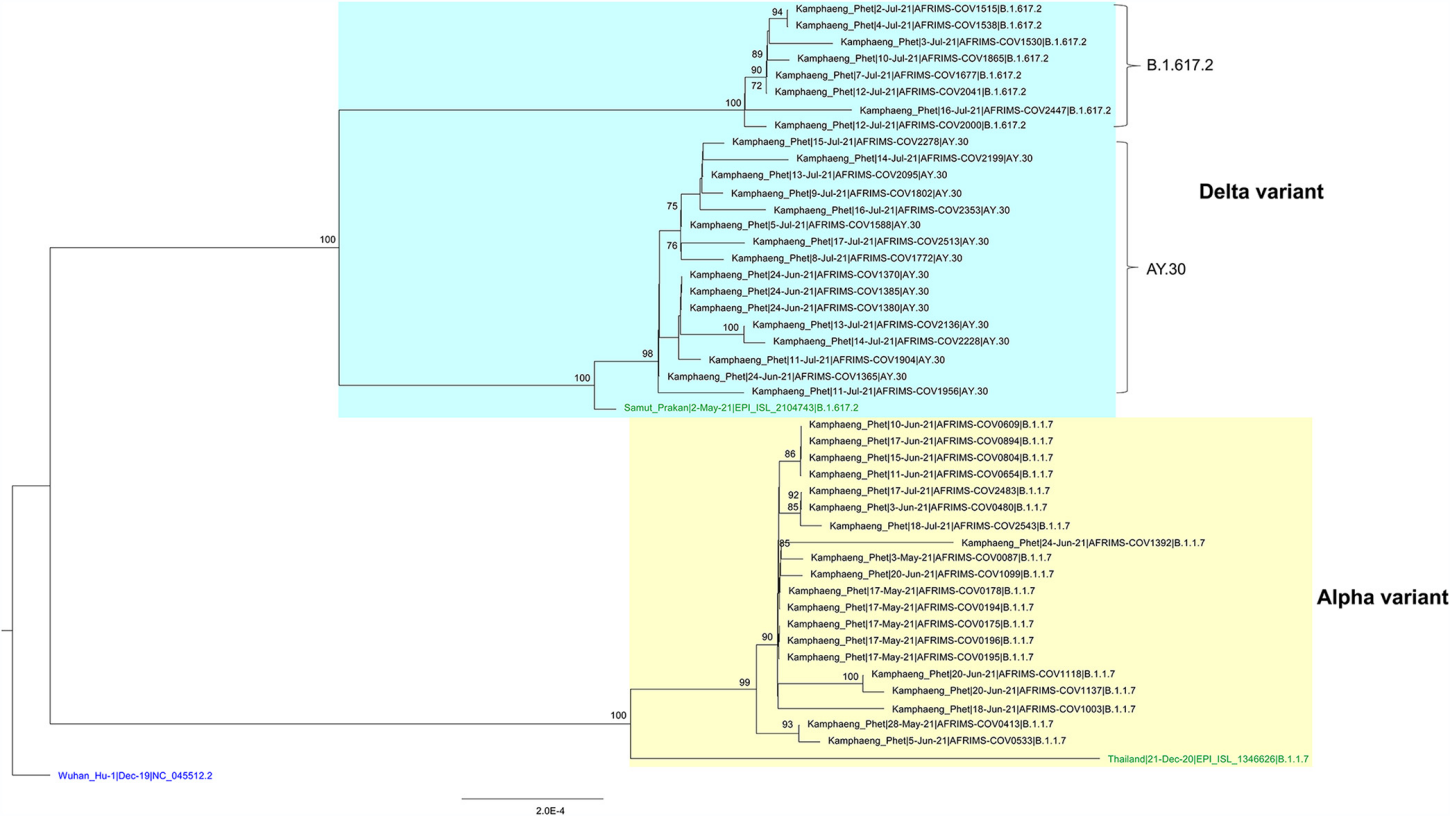
Maximum likelihood phylogenetic tree of 47 SARS-CoV-2 coding sequences, including 44 sequences from this study (black), the first collected alpha variant sequence from Thailand (GISAID accession number EPI_ISL_1346626), which was collected on 21 December 2020 (green), the first collected delta variant sequence from Thailand (GISAID accession number EPI_ISL_2104743), which was collected on 2 May 2021 (green), and the reference Wuhan-Hu-1 genome sequence (GenBank accession number NC_045512.2) (blue). Multiple sequence alignments were performed using MAFFT v7.475 with default settings (11). The tree was constructed by using IQ-TREE v2.1.2 (12) with substitution model TIM2+F+I and 1,000 ultrafast bootstrap replicates and was visualized by using FigTree v1.4.2 (13).

Individual genome characteristics are summarized in Table 1. The reads obtained were 35 to 251 nucleotides in length, and the average length was 217 nucleotides. Consensus sequences of coding regions were 29,387 to 29,402 bp in length, with the mean DOC ranging from 3,636× to 8,642×. Of 44 sequences, 20 and 24 were identified as alpha and delta variants, respectively. The alpha variants were found from 3 May 2021 to 18 July 2021, whereas the delta variants were found from 24 June 2021 to 18 July 2021. The phylogenetic tree is shown in Fig. 1.

Amino acid substitutions found in the alpha and delta variants from KPP when aligned with the sequences of the first corresponding variants collected in Thailand are shown in Table 1. Two nonsense mutations, i.e., G254* and K68* in open reading frame 3a (ORF3a) and ORF8 genes, respectively, were not found in the first alpha variant virus in Thailand but were found in all alpha variant viruses in this study. K68* was reported previously (15). G254* in ORF3a resulted in the predicted absence of 18 amino acid residues (positions 254 to 271) at the C terminus of the protein, located in a region thought to carry several B cell epitopes (16). Mutations in ORF3a were previously described as potentially having an impact on viral infectivity and pathogenesis (16–18). Among the 24 delta variant viruses from KPP, 8 sequences were identified as B.1.617.2 lineage and 16 sequences were identified as AY.30 lineage.

In conclusion, the two variants of concern, alpha and delta, were identified from May to July 2021 in KPP. Nonsense mutations in ORF3a and ORF8 were found in the alpha variant sequences. Two lineages of the delta variant were found.

### Data availability.

The sequences from this study were deposited in GenBank (accession numbers MZ888515 to MZ888557 and MZ895505). Individual accession numbers are indicated in Table 1. The raw reads were deposited in the NCBI Sequence Read Archive (SRA) (accession numbers SRR15571382 to SRR15571425). The BioProject accession number is PRJNA757144. The BioSample accession numbers are SAMN20934606 to SAMN20934649.
